# Zoonotic disease knowledge attitude and practices among rural hunting communities in Gabon

**DOI:** 10.3389/fvets.2026.1857652

**Published:** 2026-07-03

**Authors:** Serge Ely Dibakou, Natacha Efoua Tomo, Ivan Cyr Moussadji Kinga, Désiré Otsaghe Ekore, Patrice Makouloutou Nzassi, Berthe-Nadine N'dilimabaka, Roland Fabrice Kassa Kassa, Anicet Mouity Matoumba, Linaa Yasmine Okomo Nguema, Hélène Sisraëlla Lembanaka, Jenny Mathouet, Aude Pouliquen, Gilles Aurélien Boupana Mapeyi, Thierry-Audrey Tsoumbou, Barthélémy Ngoubangoye, Hadrien Vanthomme, Alexis Delabouglise, Gael Darren Maganga

**Affiliations:** 1 Unité de Bactériologie et One Health (UBOH), Centre Interdisciplinaire de Recherches Médicales de Franceville (CIRMF), Franceville, Gabon; 2 Unité 'Emergence des Maladies Virales, Département de Virologie, Centre Interdisciplinaire de Recherches Médicales de Franceville (CIRMF), Franceville, Gabon; 3 CIRAD, UMR ASTRE, Montpellier, France; 4 ASTRE, Université de Montpellier, CIRAD, INRAE, Montpellier, France; 5 Institut de Recherche Agronomique et Forestière (IRAF), Libreville, Gabon; 6 Centre de Primatologie (CDP), Centre Interdisciplinaire de Recherches Médicales de Franceville (CIRMF), Franceville, Gabon; 7 Institut de Recherches en Ecologie Tropicale (IRET), Libreville, Gabon; 8 Unité de Recherches en Ecologie de la Santé (URES), Centre Interdisciplinaire de Recherches Médicales de Franceville (CIRMF), Franceville, Gabon; 9 Département de Biologie, Université des Sciences et Techniques de Masuku (USTM), Franceville, Gabon; 10 Laboratoire d'Analyse Médicales, Centre Interdisciplinaire de Recherches Médicales de Franceville (CIRMF), Franceville, Gabon; 11 CIRAD, UPR Forêts et Sociétés, Montpellier, France; 12 Forêts et Sociétés, Université de Montpellier, Cirad, Montpellier, France; 13 Institut d'Agronomie et de Biotechnologies (INSAB), Université des Sciences et Techniques de Masuku (USTM), Franceville, Gabon

**Keywords:** attitudes and practices, bushmeat hunting, Gabon, knowledge, One Health, rural communities, spillover risk, zoonotic diseases

## Abstract

**Introduction:**

Zoonotic diseases represent a major public health concern in Central Africa, where forest-dependent communities experience frequent contact with wildlife through hunting activities, bushmeat handling, consumption, and trade. This study assessed knowledge, attitudes, and practices related to zoonotic diseases among communities engaged in hunting practices, bushmeat consumption, and wildlife trade in Mulundu Department.

**Methods:**

A cross-sectional questionnaire-based survey was conducted from April 2023 to April 2024 among 326 participants from five communities. Data on sociodemographic characteristics, awareness of zoonoses, perceived transmission routes, clinical signs, attitudes, and preventive practices were collected through structured interviewer-administered questionnaires. Knowledge was assessed using 13 items and categorized into poor or good knowledge level. Associations between knowledge level and participant characteristics were examined using Pearson's chi-square tests and logistic regression.

**Results:**

Overall, 72.7% of respondents had previously heard of zoonotic diseases, with Ebola being the most frequently identified. Most participants had poor knowledge overall (69.3%), while 30.7% had good knowledge. Knowledge level varied significantly by regroupement (*p* = 0.037), and age (*p* = 0.023). Attitudes toward prevention were generally positive, up to 31.0% of participants remained uncertain about how to respond appropriately in high-risk situations.

**Discussion:**

These findings highlight important geographic and behavioral gaps in zoonotic disease preparedness and support the need for sustained, culturally adapted, community-based One Health interventions in rural communities in Gabon.

## Introduction

Zoonotic diseases, caused by pathogens naturally transmitted from animals to humans, represent a persistent and growing threat to global public and veterinary health (1, 2). They constitute the primary driver of Emerging Infectious Diseases (EIDs), with historical trends indicating that over 75% of EIDs originate from wildlife reservoirs (3–8). The emergence and re-emergence of these pathogens are often intricately linked to large-scale anthropogenic change, including deforestation, habitat destruction, climate changes, and agricultural intensification. These factors increase interactions among humans, domestic animals, and wildlife, thereby raising the risk of pathogen spillover (6). Zoonotic diseases are classified based on etiology, transmission cycle, reservoir hosts, pathogenesis, and primary human symptoms. Notable examples include Ebola virus disease, Middle East respiratory syndrome (MERS), COVID-19, Lyme disease, anthrax, bovine tuberculosis, zoonotic malaria, yellow fever, swine flu, avian influenza, rabies, and monkeypox (Mpox) (9).

Sub-Saharan Africa is recognized as a hotspot for zoonotic EIDs, where high wildlife biodiversity, rapid environmental change, and frequent human-animal interactions converge to create ideal conditions for spillover risk (10, 11). The Central African region, particularly the Congo Basin with its dense rainforests and abundant wildlife, serves as a critical epicenter for EID emergence (12–14). This region has played a central role in the emergence, maintenance, or spillover of several pathogens with substantial global health consequences, including Ebola viruses, Monkeypox virus (MPXV), and simian retroviruses that successfully crossed into humans and evolved into human immunodeficiency viruses (HIV-1 and HIV-2) (14–18). Moreover, hunting practices, bushmeat consumption, and wildlife trade in this region intensify human-wildlife contact and increase exposure to wildlife-associated pathogens, thereby heightening the risk of zoonotic spillover and pandemic emergence (5).

In Gabon, where approximately 80% of the land is forested, rural communities rely heavily on hunting bushmeat, fishing, and gathering for subsistence and income. This reliance is deeply rooted in local culture and economy, with wild meat serving as an essential source of protein and a vital component of food security for households in remote and economically vulnerable areas (14–19). These livelihood activities involve frequent and close contact between humans and wildlife. The highest risks of zoonotic pathogen spillover arise during hunting, butchering, and preparation of wild animals, which entails direct exposure to infectious materials such as blood, tissues, and bodily fluids (13, 20). Addressing these high-risk behaviors remains particularly challenging, as their persistence is shaped by a complex combination of economic necessity, food insecurity, limited access to affordable alternative protein sources, cultural traditions, taste preferences, social norms, and the deep-rooted role of hunting in local identity and community life (1).

Preliminary findings from Central Africa, including Gabon, suggest that perception of zoonotic risks is generally low and biased. The immediate economic benefits of consuming or selling animals, including those found dead or exhibiting signs of disease, strongly limit the adoption of safer practices (1, 21). This discrepancy between latent knowledge and actual behavior is exemplified by a study in rural Nigeria where 55% of hunters recognized the risk of contracting diseases from wildlife, yet only 26% implemented precautions during slaughter (15). In this context, knowledge of zoonotic risk does not necessarily translate into protective behavior because risk perception is filtered through everyday experience, cultural familiarity with wildlife, perceived necessity, and locally grounded ideas about what constitutes safe or unsafe meat. Despite their significance for risk mitigation, comprehensive quantitative data on Knowledge, Attitudes and Practices (KAP) regarding zoonoses among forest-dependent communities in Central Africa, particularly in Gabon, remain scarce (1). Existing risk assessments in Gabon confirm low risk perception among wild meat consumers and producers and suggest that behavioral decisions are strongly shaped by factors such as alternative sources of livelihoods and trust in sanitary authority (21). Previous studies among smallholder farmers have shown that KAP assessments can reveal critical gaps in awareness and risky practices, such as inadequate biosecurity, unsafe handling of animal products and limited understanding of transmission pathways (22–25). However, the specific risks, behaviors and economic drivers associated with wildlife-related zoonoses differ substantially from those observed in conventional livestock production systems (26). While studies of livestock production systems often focus on farm management practices, vaccination, and biosecurity measures, the risks associated with wildlife-related zoonoses are more closely linked to hunting, handling bushmeat, and informal value chains (25, 26).

Conducting a KAP study within these communities constitutes a public health imperative. Such a study will identify knowledge gaps and misconceptions regarding zoonotic disease transmission. It will also elucidate how local attitudes, cultural beliefs and risk perceptions may facilitate or hinder disease prevention efforts. Additionally, it will document routine practices, both high-risk and protective, occurring at the human-wildlife interface. Generating this empirical evidence is essential to move beyond qualitative observation and to establish a foundation for targeted, culturally appropriate, and effective One Health interventions. Accordingly, the present study aims to assess knowledge, attitudes, and practices regarding zoonotic diseases among communities engaged in hunting practices, bushmeat consumption, and wildlife trade in Mulundu Department.

## Materials and methods

### Description of the study area

This cross-sectional study was conducted from April 2023 to April 2024 in Mulundu Department, which is located in the Ogooué-Lolo Province, in Gabon. This rural region is characterized by dense equatorial forests. Livelihoods in this region are largely based on subsistence agriculture, hunting, and small-scale trade, resulting in frequent interactions between humans, domestic animals, wildlife, and the surrounding environmental conditions that are particularly relevant for zoonotic disease transmission within a One Health context. Wildlife hunting occurs year-round and constitutes a major source of dietary protein and income for rural communities. Hunting activities are regulated and the main species hunted, including duikers, porcupines, red river hogs, and monkeys, are not currently classified as endangered based on ecosystem productivity assessments (27). The study population consisted of communities involving in the EU-funded Sustainable Wildlife Management (SWM) Program (https://www.fao.org/in-action/swm-programme). The SWM Program is a multi-country initiative (active in 15 African, Caribbean, and Pacific countries) that works to improve wildlife conservation and food security. In Gabon, the program focuses on developing community-based management plans for wild meat hunting, establishing sustainable harvest quotas based on ecosystem productivity assessments, and creating legal supply chains for wild meat. The program operates through a consortium including the Food and Agriculture Organization of the United Nations (FAO), the French Agricultural Research Centre for International Development (CIRAD), the Centre for International Forestry Research (CIFOR) and the Wildlife Conservation Society (WCS). Within the framework of the SWM Program, the Centre Interdisciplinaire de Recherches Médicales de Franceville (CIRMF) implemented a community-based surveillance initiative for zoonotic diseases in the Mulundu Department. The initiative involved: (1) training community members as One Health sentinels to identify and report potential warning signs of zoonotic disease in both wildlife and humans, including unusual animal mortality, abnormal animal behavior, visible lesions, and clusters of febrile illness; (2) establishing a mobile phone-based reporting system for unusual animal deaths or human illness clusters between sentinels, community focal points, and CIRMF teams; (3) monthly community meetings to review reported events, discuss surveillance findings, reinforce risk awareness, and promote safer practices; and (4) quarterly biological sampling of hunted animals, including blood and tissue collection for laboratory screening at CIRMF.

### Study population and eligibility criteria

The study population consisted of residents from five administrative units of the department, hereafter anonymized as Regroupements A–E. These five communities were selected to capture variation in geographical accessibility, socioeconomic conditions, wildlife exposure, livelihood practices, and access to health and communication infrastructure. Accordingly, the sample included regroupements located along accessible road networks, where movement, trade, communication, and access to health services are relatively facilitated, as well as more remote settlements connected primarily by forest tracks, where limited transport options and mobile network coverage may constrain timely access to care, information, and surveillance reporting. Individuals were eligible to participate if they were residents of one of the selected regroupements, and were present during the survey period. In addition, participant recruitment followed a field-based voluntary enrolment approach rather than a strictly probabilistic sampling strategy. This approach was adopted because the study was embedded within ongoing community-based zoonotic disease awareness and surveillance activities.

### Sample size

The minimum sample size required for this KAP study was estimated using Cochran's formula for proportions: *n0* = *Z*^2^*pq/d*^2^, where *Z* is the standard normal deviate corresponding to a 95% confidence level, *p* is the expected proportion of participants with adequate knowledge of zoonotic diseases, *q* = *1–p*, and *d* is the desired margin of error. Because no prior KAP data were available for rural hunting communities in Mulundu Department, an expected proportion of 50% was used to maximize the required sample size. Using *Z* = *1.96, p* = *0.50, q* = *0.50*, and *d* = *0.05*, the initial sample size was estimated at 384.16 and rounded up to 385 participants. Given that the target population of Mulundu Department is finite, estimated at 27,750 inhabitants (28), a finite population correction was applied using the formula *n* = *n0/[1* + *(n0 – 1)/N]*, where *N* represents the total population size. This adjustment yielded a corrected minimum sample size of approximately 379 participants. In the present study, 326 eligible and available participants were enrolled during the data-collection period across the five participating regroupements. Although the final sample size was below the corrected minimum estimated sample size, it was considered acceptable for an operational community-based study conducted under rural field conditions. Nevertheless, this shortfall is acknowledged as a methodological limitation.

### Study design and data collection

A questionnaire-based KAP cross-sectional study was conducted to assess the knowledge, attitude, and prevention practices concerning zoonotic diseases among community members of the five regroupements. Prior to the implementation of the study, village leaders from all regroupements were informed about the study objectives and procedures by teams from the SWM Program, CIRAD, as part of the rollout of a zoonotic disease surveillance initiative led by CIRMF. Community sensitization was conducted during routine field visits, which facilitated participant mobilization and engagement during the training sessions and questionnaire administration.

Data were collected using a structured French-language questionnaire administered to participants in four main survey sessions corresponding to pre- and post-training assessment (Table 1). The initial study design sought to re-interview the same participants across both assessment periods in order to evaluate changes in KAP indicators over time. However, field constraints, participant mobility, and variations in community availability limited complete individual-level matching between the two survey rounds. Consequently, pre- and post-training assessments were treated as independent observations in the statistical analyses. The questionnaire included both closed- and open-ended questions covering socio-demographic characteristics, knowledge of zoonotic diseases, risk perceptions of zoonotic transmission, attitudes toward wildlife-associated health risks, and preventive practices related to hunting practices, animal handling, bushmeat consumption, and healthcare-seeking behavior (Supplementary material). Face-to-face interviews were conducted by trained field investigators in the participants' communities, with each interview lasting approximately 25–35 min. When participants were not comfortable responding in French, questions were explained orally in the locally understood language by trained interviewers using standardized wording agreed in accordance with village leaders before data collection. This approach was used to reduce literacy-related exclusion and to improve comprehension.

**Table 1:** Pre- and post-training questionnaire administration conducted to assess changes in knowledge, attitudes, and practices related to zoonotic diseases following community-based training interventions.

Study phase	Period	Regroupements	Questionnaire administration	Training session	Notes
Phase 1 (Baseline)	April 2023	Regroupement B, Regroupement E, Regroupement D	Pre-training (baseline)	Conducted after questionnaire	Training covered zoonotic diseases including awareness, transmission routes, symptoms in humans and animals, risk perception, and prevention); interactive and context-adapted
Phase 1 (evaluation phase)	June 2023	Regroupement B, Regroupement E	Post-training	No	Regroupement D not surveyed due to participant unavailability (agricultural activities and death of village chief)
Phase 2 (Baseline)	November 2023	Regroupement A, Regroupement C	Pre-training (baseline)	Conducted after questionnaire	Same standardized training content as Phase 1
Phase 2 (evaluation phase)	April 2024	Regroupement A, Regroupement C	Post-training	No	Follow-up conducted in same regroupements

### Knowledge level and classification

Knowledge of zoonotic diseases was assessed using 13 questionnaire items (Supplementary material). A correct answer was coded with a score of 1 point while an incorrect or “I do not know” answer was coded with 0 points. A total knowledge score was then computed by summing the 13 items, yielding a continuous score ranging from 0 to 13, with higher values indicating greater knowledge of zoonotic diseases. This score was then transformed into a percentage reflecting overall performance with 100% representing perfect alignment with the desired knowledge. Based on the widely adopted Bloom's cut-off, the knowledge was categorized as follow: 80%−100% (good), 60%−79% (moderate) and less than 60% (poor) (29–31). Applied to the 13-point scale used in this study, these thresholds corresponded to poor knowledge scores of 0–7, moderate knowledge scores of 8–10, and good knowledge scores of 11–13. In this study, a modified Bloom's cut-off value was used to categorize the knowledge of participants into two levels (30, 31). Participants with a knowledge score from 0 to 8 were classified as having poor knowledge, whereas participants with a knowledge score **≥** 9, were classified as having good knowledge.

### Ethics approval and consent to participate

Ethical approval for this study was obtained from the Gabonese National Ethics Committee for Research in Public Health (Comité National d'Éthique en matière de Recherche Scientifique (CNERS) of Gabon; n°0021/2024/MESRSIT/CNERS/PR/SG). The collection, management, and use of personal data were conducted in accordance with the European Union General Data Protection Regulation (GDPR; Regulation 2016/679). Participation was entirely voluntary. Before questionnaire administration, trained field staff explained the study objectives, procedures, confidentiality measures, voluntary nature of participation, and participants' right to decline or withdraw at any time without consequence. Given variation in literacy levels across communities, verbal informed consent was obtained prior to participation, in accordance with the approved ethics protocol. For the post-training assessment, participants' willingness to continue or renew participation was confirmed before administering the questionnaire. All data were collected anonymously, stored securely, and handled confidentially throughout the study.

### Data analysis

Data obtained from the questionnaires were entered into Microsoft Excel, cleaned, coded, and exported to R software version 4.4.0 for statistical analysis. Descriptive statistics were performed to summarize the sample sociodemographic characteristics and evaluate the overall distribution in KAP variables. Pearson's chi-squared test was used to assess the association between participants' sociodemographic characteristics and their knowledge level, followed by logistic regression to determine the relative importance of selected sociodemographic variables in predicting participants' knowledge level. The significance level was set at *p* < 0.05.

## Results

### Demographic characteristics of the participants

The questionnaire was administered to 326 respondents, including 55.2% males and 44.8% females. Most respondents were aged 45 years or older (51.5%), followed by 30–45 years (20.2%), 19–29 years 18.7%, and under 19 years 9.5%. Educational attainment was generally low: 12.8% were illiterate, 39.0% had primary education, and 47.0% had completed secondary education. Only four participants (1.2%) reported having a university degree. The predominant occupations were farming (39.0%), hunting (16.9%), and formal employment (14.1%) (Table 2).

**Table 2:** Demographic characteristics of the study participants.

Demographic characteristic	Frequency (*N* = 326)	%
Sex		
Male	180	55.2
Female	146	44.8
Age (years)		
<19	31	9.51
19–29	61	18.71
30–45	66	20.25
>45	168	51.53
Education level		
Illiterate	42	12.8
Primary	127	39.0
Secondary	153	47.0
University	4	1.2
Occupation status		
Farmer	127	39.0
Hunter	55	16.9
Salaried worker	46	14.1
Student	30	9.2
Retired	20	6.1
Trader	10	3.1
Healer	4	1.2
Driver	1	0.3
Job seeker	33	10.1
Residence		
Regroupement E	59	18.1
Regroupement B	119	36.5
Regroupement D	50	15.34
Regroupement C	47	14.42
Regroupement A	51	15.64

### Knowledge of zoonotic diseases among the community

#### General awareness and diseases recognition

Among respondents, 72.7% (*n* = 237) reported prior awareness of zoonotic disease. Ebola was the most commonly known zoonosis (56.1%), followed by rabies (16.6%), and COVID-19 (5.0%). Tuberculosis, Marburg virus, and parasitic infections were less frequently cited (Table 3).

**Table 3:** Participants' responses related to general knowledge about zoonoses (*N* = 326).

Knowledge questions	Response	Frequency	Percentage (%)
Hear about zoonotic disease	Yes	237	72.7
	No	89	27.3
Type of zoonotic disease	COVID-19	16	5.0
	Ebola	183	56.14
	Marburg	6	1.8
	Parasitic zoonoses	2	0.6
	Rabies	54	16.6
	Tuberculosis	11	3.4
Hear about animals responsible for zoonotic disease	Yes	222	68.1
	No	104	31.9
Type of animal species responsible for zoonotic disease	Bats	42	12.9
	Dogs	62	19.0
	Pangolin	30	9.2
	Non-human Primate	159	48.8
	Rodents	13	4.0
	Duiker	6	1.8
	Porcupine	6	1.8
Transmission routes of zoonoses	Scratching	164	50.3
	Biting	195	59.8
	Licking	90	27.6
	Handling animals	187	57.4
	Consuming contaminated meat	165	50.6
	Eating infected organs	105	32.2
	Contaminated water	150	46.0
	Inhalation	84	25.8
Zoonotic diseases can kill	Yes	258	79.1
	No	68	20.9
Know signs of zoonotic disease in humans	Fatigue	13	4.0
	Fever	38	11.6
	Weight loss	29	9.0
	Diarrhea	44	13.5
	Vomiting	14	4.2
	Haemorrhagy	6	2.0
Know signs of zoonotic disease in animals	Fatigue	25	7.6
	Fever	2	0.6
	Weight loss	61	18.7
	Diarrhea	6	1.8
	Vomiting	4	1.2
	Haemorrhagy	9	2.7

#### Awareness of animal reservoirs

Approximately 68.1% of participants identified at least one animal species associated with zoonotic disease transmission. Non-human primates were the most frequently cited (48.8%), followed by dogs (19.0%), bats (12.9%), and pangolins (9.2%). Rodents, duikers, and porcupines were rarely mentioned (Table 3).

#### Knowledge of transmission routes

Participants showed varying knowledge levels of transmission pathways. The most commonly recognized routes were animal bites (59.8%), handling infected animals (57.4%), scratches (50.3%), and consumption of contaminated meat (50.6%). Fewer respondents identified contaminated water (46.0%), eating infected organs (32.2%), or inhalation (25.8%) as transmission risks (Table 3).

#### Knowledge of clinical symptoms

Awareness of clinical symptoms in both humans and animals was low. For human illness, 13.5% named diarrhea, 11.6% identified fever, and 9.0% cited weight loss. For animals, 18.7% recognized weight loss as a sign of disease, while fewer than 10% mentioned fatigue, diarrhea, or hemorrhage (Table 3).

### Factors associated with knowledge of zoonotic diseases

**Figure 1:**
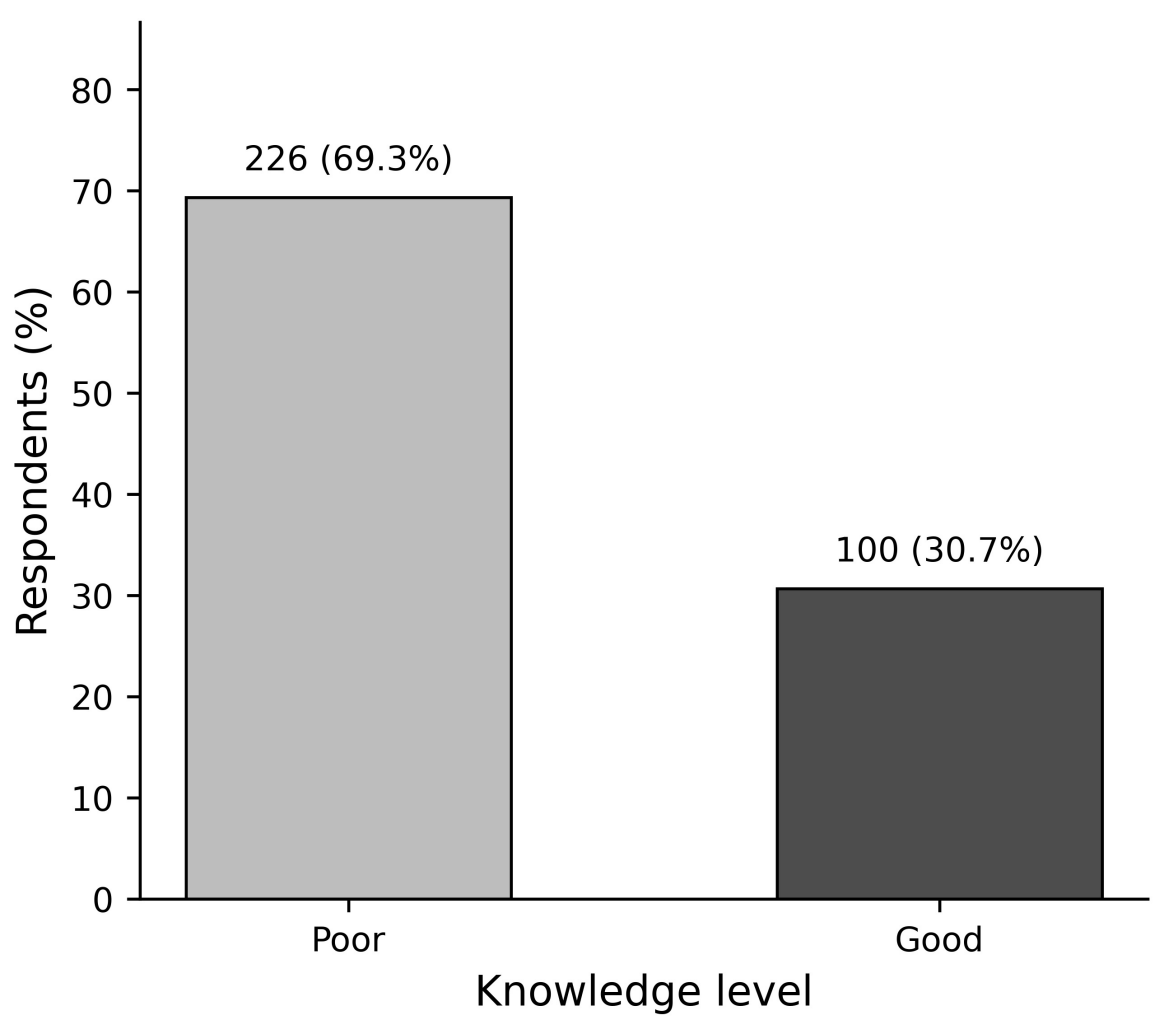
Knowledge level distribution across the study population (*N* = 326). Bars show the percentage of respondents classified as having poor or good knowledge; labels shown (%).

**Figure 2:**
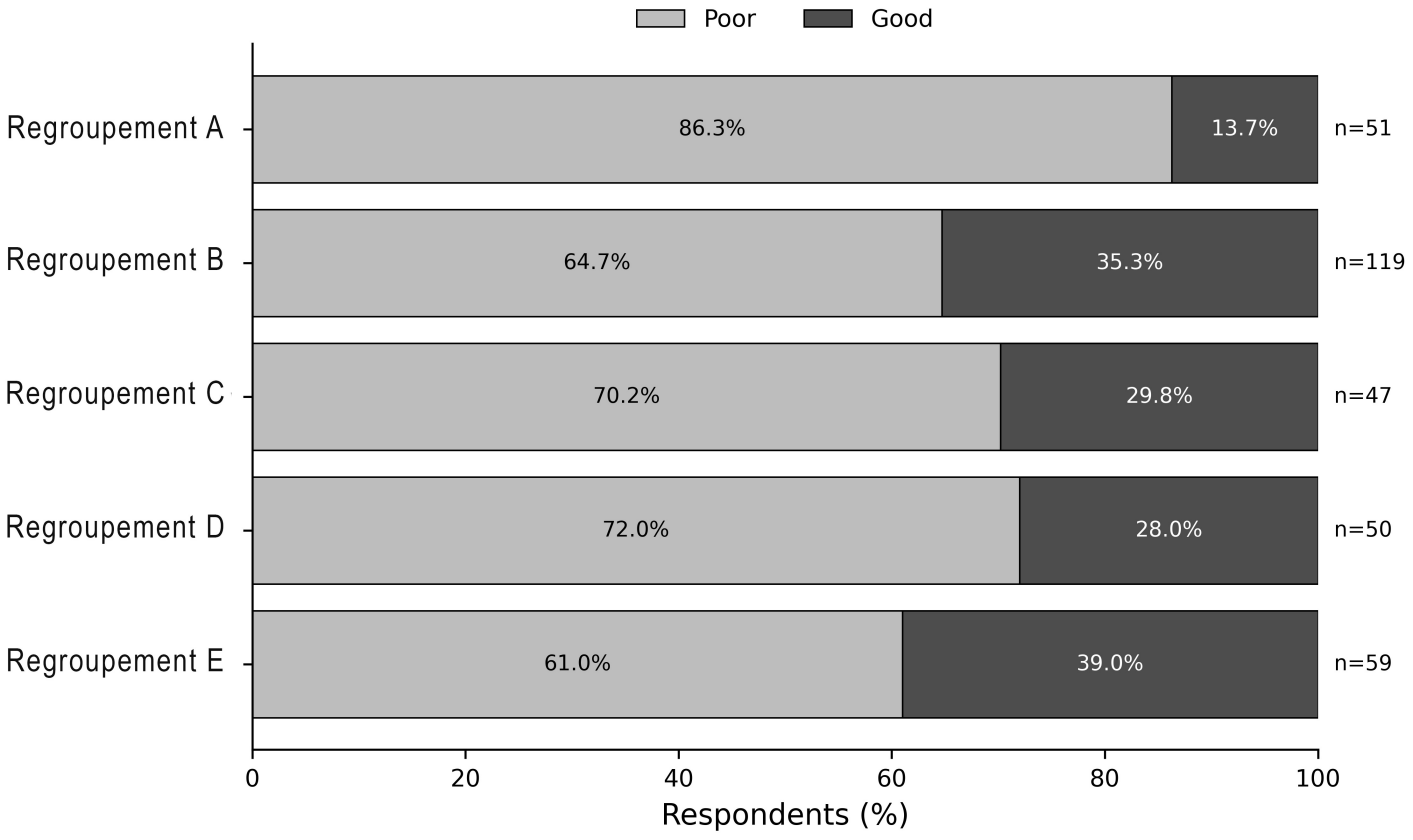
Knowledge level distribution across regroupement (*N* = 326). Horizontal stacked bars show within-regroupement percentages of poor and good knowledge; *n* values indicate the number of respondents per regroupement.

Overall, 226 participants (69.3%) had a poor knowledge level, while 100 respondents (30.7%) had a good knowledge level (Figure 1). Bivariate analyses showed that only regroupement was significantly associated with knowledge level (*p* = 0.037). The proportion of participants with good knowledge was highest in Regroupement E (23/59, 39.0%) and Regroupement B (42/119, 35.3%), and lowest in Regroupement A (7/51, 13.7%). No significant association was observed among the other variables examined (Figure 2, Table 4).

**Table 4:** Association between knowledge level and socio-demographic characteristics of respondents (*N* = 326).

Demographic characteristic	Knowledge level		*p*-value
	Poor *n* (%)	Good *n* (%)	
Sex			
Female	101 (69.2)	45 (30.8)	0.958
Male	125 (69.4)	55 (30.6)	
Age group			
<19	22 (71.0)	9 (29.0)	0.125
19–29	40 (65.6)	21 (34.4)	
30–45	39 (59.1)	27 (40.9)	
>45	125 (74.4)	43 (25.6)	
Education level			
Illiterate	31 (73.8)	11 (26.2)	0.506
Primary	86 (68.3)	40 (31.7)	
Secondary	105 (68.2)	49 (31.8)	
University	4 (100.0)	0 (0.0)	
Occupation status			
Farmer	88 (69.3)	39 (30.7)	0.664
Hunter	42 (76.4)	13 (23.6)	
Salaried worker	30 (65.2)	16 (34.8)	
Student	22 (73.3)	8 (26.7)	
Retired	13 (65.0)	7 (35.0)	
Trader	6 (60.0)	4 (40.0)	
Healer	4 (100.0)	0 (0.0)	
Driver	1 (100.0)	0 (0.0)	
Job seeker	20 (60.6)	13 (39.4)	
Residence			
Regroupement E	36 (61.0)	23 (39.0)	**0.037** ^*^
Regroupement B	77 (64.7)	42 (35.3)	
Regroupement D	36 (72.0)	14 (28.0)	
Regroupement C	33 (70.2)	14 (29.8)	
Regroupement A	44 (86.3)	7 (13.7)	
Assessment			
POST	87 (65.9)	45 (34.1)	0.269
PRE	139 (71.6)	55 (28.4)	

^*^P < 0.05. Pearson's chi-square test was used. Bold values indicate statistical significance at *p* < 0.05.

**Figure 3:**
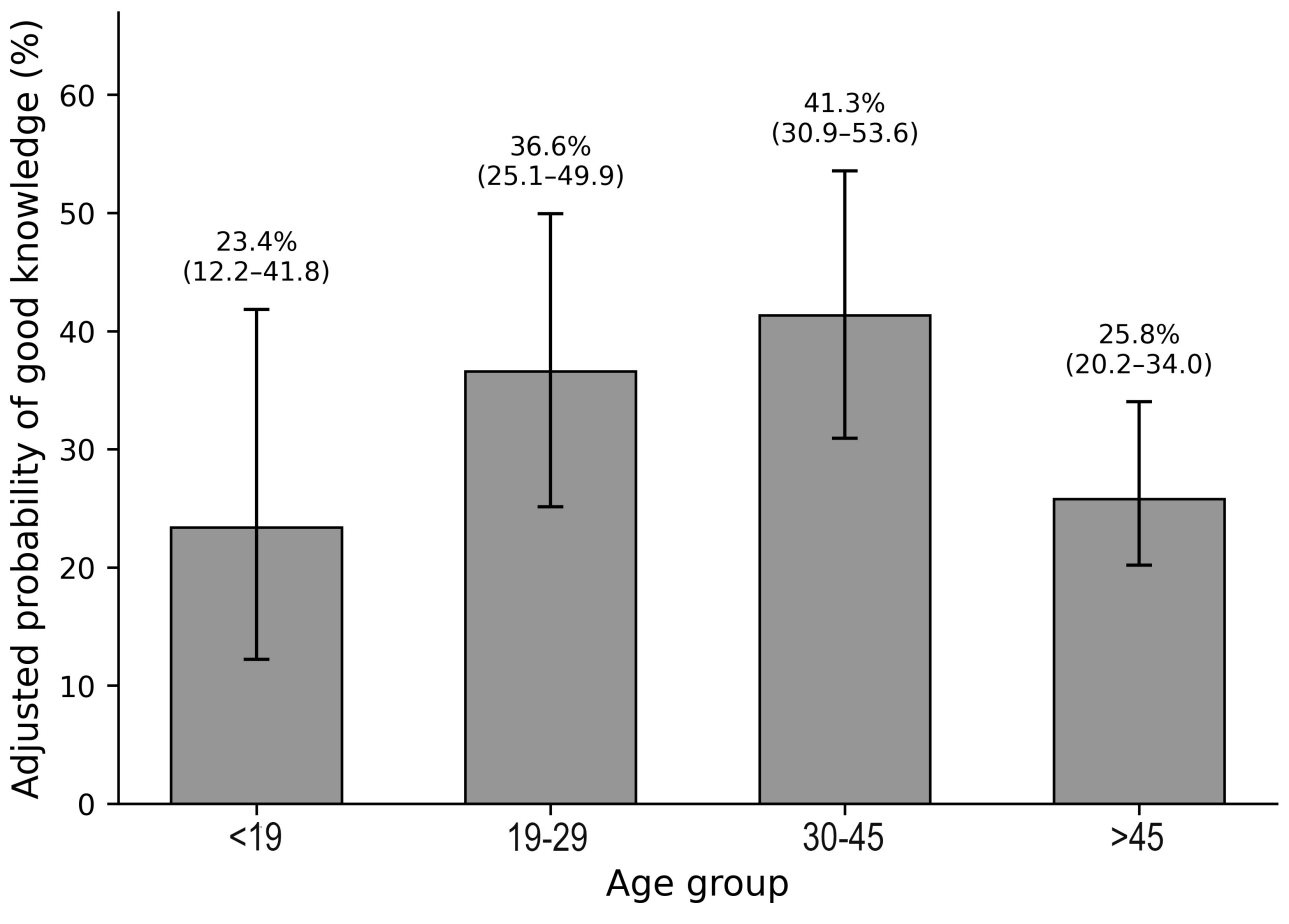
Adjusted probability of good knowledge by age group (*N* = 326). Bars show marginal predicted probabilities from a multivariable logistic regression model adjusted for assessment, regroupement, sex, education level, and occupation. Error bars show 95% confidence intervals. Education and occupation were grouped to avoid sparse-cell model instability.

Logistic regression analyses showed two main results. After adjustment for other participant characteristics, regroupement, and age group were significantly associated with knowledge level. Participants from Regroupement A had significantly lower odds of good knowledge compared with those from Regroupement B **(**aOR = 0.35, 95% CI: 0.14–0.91, *p* = 0.031). Conversely, adult respondents had significantly higher odds of good knowledge compared with elderly respondents (aOR = 2.20, 95% CI: 1.11–4.36, *p* = 0.023) (Figure 3, Table 5).

**Table 5:** Logistic regression analysis between knowledge level and respondent characteristics (*N* = 326).

Demographic characteristic	Knowledge level		*p-value*
	OR (95% CI)	aOR (95% CI)	
Sex			
Female	Reference	Reference	—
Male	0.99 (0.62–1.59)	1.45 (0.77–2.75)	0.252
Age group			
<19	1.19 (0.51–2.78)	1.27 (0.32–5.07)	0.735
19–29	1.53 (0.81–2.87)	1.73 (0.79–3.78)	0.168
30–45	2.01 (1.10–3.67)	**2.20 (1.11–4.36)**	**0.023** ^*^
M>45	Reference	Reference	—
Education level			
Illiterate	Reference	Reference	—
Primary	1.31 (0.60–2.87)	1.53 (0.66–3.57)	0.324
Secondary	1.32 (0.61–2.83)	1.12 (0.48–2.62)	0.795
MUniversity	Not estimable	Not estimable	—
Occupation status			
Farmer	Reference	Reference	—
Hunter	0.70 (0.34–1.45)	0.62 (0.26–1.48)	0.282
Salaried worker	1.20 (0.59–2.46)	0.73 (0.31–1.72)	0.475
Student	0.82 (0.34–2.00)	0.61 (0.14–2.60)	0.503
Retired	1.21 (0.45–3.28)	1.19 (0.37–3.85)	0.769
Trader	1.50 (0.40–5.63)	0.98 (0.24–3.97)	0.972
Healer	Not estimable	Not estimable	—
Driver	Not estimable	Not estimable	—
MJob seeker	1.47 (0.66–3.24)	1.26 (0.52–3.07)	0.607
Residence			
Regroupement E	1.17 (0.61–2.23)	1.37 (0.66–2.81)	0.399
Regroupement B	Reference	Reference	—
Regroupement D	0.71 (0.35–1.47)	0.79 (0.34–1.84)	0.586
Regroupement C	0.78 (0.38–1.61)	0.83 (0.37–1.89)	0.658
Regroupement A	0.29 (0.12–0.70)	**0.35 (0.14–0.91)**	**0.031** ^*^
Assessment			
POST	1.31 (0.81–2.11)	1.29 (0.74–2.26)	0.366
PRE	Reference	Reference	—

OR is unadjusted OR from univariate logistic regression analysis.

aOR is adjusted OR or OR that has been adjusted for all other variables from multivariate logistic regression analysis.

*P*-value shown in the table is from the adjusted logistic regression model. ^*^P < 0.05.

Reference = reference category used for comparison in the logistic regression model; Not estimable = odds ratio could not be estimated because of sparse data.

– = not applicable.

Bold values indicate statistical significance at *p* < 0.05.

### Knowledge according to pre- and post-training questionnaire administration

Knowledge level did not differ significantly between the pre-training and post-training assessment phases (*p* = 0.269), although the proportion classified as having good knowledge was numerically higher after training (Table 4). Good knowledge was observed in 55 of 194 participants (28.4%) in the pre-training phase and 45 of 132 participants (34.1%) in the post-training phase, whereas poor knowledge was observed in 71.6% and 65.9% of participants, respectively.

### Attitude toward zoonotic disease prevention

Responses to hypothetical risk scenarios revealed generally positive attitudes despite some uncertainty: when asked how they would respond if a loved one appeared ill, 50.3% indicated they would seek hospital care; 8.3% would notify authorities, and 31.0% were uncertain or did not know how to respond (Table 6). In a hypothetical scenario involving a sick animal, 34.0% would avoid contact, 13.8% would alert authorities, and 9.5% reported they would kill the animal (Table 6). Similarly, upon finding a dead animal, 46.0% would avoid it, whereas 2.5% indicated they might consume the carcass (Table 6). Finally, if they themselves became ill from a zoonosis, 59.2% would seek hospital care, 30.4% were uncertain, and 2.8% would use traditional medicine (Table 6).

**Table 6:** Attitude related responses among participants (*N* = 326).

Attitude variables	Response	Frequency	Percentage (%)
If a loved one shows signs of illness	Go to the hospital	164	50.3
	Don't know	101	31.0
	Alert authorities	27	8.3
	Avoid contact	15	4.6
	Quarantine	10	3.1
	Traditional medicine	7	2.1
	Go to the church	2	0.6
If you see a sick animal	Don't know	119	36.5
	Avoid contact	111	34.0
	Alert authorities	45	13.8
	Kill	31	9.5
	Treat	6	1.8
	Do nothing	5	1.5
	Go to the hospital	4	1.2
	Quarantine	4	1.2
	Veterinary medicine	1	0.3
If you find a dead animal	Avoid contact	150	46.0
	don't know	106	32.5
	Alert authorities	32	9.8
	Bury	24	7.4
	Eat	8	2.5
	Veterinary medicine	6	1.8
If you catch an animal-borne disease	Go to the hospital	193	59.2
	don't know	99	30.4
	Alert authorities	17	5.2
	Traditional medicine	9	2.8
	Quarantine	3	0.9
	Avoid contact	3	0.9
	Go to the church	2	0.6

### Reported preventive practices and risk factors

Participants showed a high reliance on modern medicine (80.7%); however, a substantial proportion also expressed strong belief in traditional or spiritual protection. Specifically, more than half (54.6%) believed that prayer could protect against zoonotic diseases, while 39.0% endorsed the protective value of traditional medicine. In addition, 35.0% of participants believed that herbal baths offered protection. In contrast, belief in protective charms was relatively uncommon, reported by only 8.0% of respondents (Table 7).

**Table 7:** Practices related responses among participants (*N* = 326).

Practice/Perception variable	Response	Frequency	Percentage (%)
Prayer can protect you from zoonotic diseases	Yes	178	54.6%
	No	148	45.4%
Charm can protect you from zoonotic diseases	Yes	26	8.0%
	No	300	92.0%
Traditional bath can protect you from zoonotic diseases	Yes	114	35.0%
	No	212	65.0%
Traditional medicine can protect you from zoonotic diseases	Yes	127	39.0%
	No	199	61.0%
Modern medicine can protect you from zoonotic diseases	Yes	263	80.7%
	No	63	19.3%

## Discussion

With 326 responses gathered from all 5 regroupements from Mulundu, this study's findings revealed the general awareness of zoonotic diseases, as well as knowledge, risk perceptions, attitudes, and preventive practices. Overall, the majority of participants (72.7%) reported that they had heard of zoonotic diseases. This was relatively satisfactory when compared with findings from other countries including Rwanda (50.3%) (32), India (80%) (2), Burkina Faso (58.7%) (33), Ethiopia (90.34%) (34), and Vietnam (61%) (35).

Ebola was the zoonotic disease most frequently identified by respondents, likely due to its high mortality and notable local outbreaks, particularly those affecting neighboring provinces in Gabon and the Republic of Congo (1, 36). By contrast, awareness of rabies, tuberculosis, and COVID-19 was lower than might be expected given their broader public health relevance. Similar contrasts have been reported elsewhere, where diseases associated with major outbreaks or intense media coverage tend to dominate community awareness, while other endemic or less visible zoonoses receive less attention (22, 32, 37, 38). In the present study, knowledge of reservoir species also appeared selective. Non-human primates and dogs were more frequently mentioned than bats or rodents, even though the latter are important in the ecology of several zoonotic pathogens in Africa. This pattern is consistent with findings from other settings showing that community knowledge tends to be shaped by visible animals, locally salient disease narratives, and lived experience rather than by a broader understanding of reservoir ecology (39). The same pattern was evident for transmission routes and symptom recognition. Respondents more often recognized direct routes of transmission, such as animal bites, handling sick animals, and consuming bushmeat than foodborne, waterborne, or environmental pathways, and recognition of clinical signs in both humans and animals remained particularly weak. Similar knowledge gaps have been described in Ethiopia (25, 34), Uganda (40), and Vietnam (26), where communities often recognize obvious animal contact risks but are less familiar with indirect exposure pathways or early clinical warning. From a prevention perspective, this is important because delayed recognition of symptoms in animals or humans may reduce opportunities for timely reporting or risk mitigation. In communities where hunting, butchering, food preparation, and environmental exposure are routine parts of daily life, limited symptom recognition may be especially relevant for both household-level decision-making and community-based surveillance.

Multiple demographic and contextual factors influenced respondents' knowledge of zoonotic risk. A key finding of this study was the significant association between knowledge level and regroupement. Respondents from Regroupement A were significantly less likely to have good knowledge than those from Regroupement B, both in the Chi-square analysis and after adjustment for other respondent characteristics. This suggests that knowledge gaps were not uniformly distributed across the study area but were clustered geographically. Such differences may reflect variation in previous exposure to health education, access to public health services, local communication networks, proximity to awareness campaigns, or differences in livelihood activities involving animals. Geographic heterogeneity in zoonotic disease knowledge has been reported in Ethiopia (34) and in Nepal (41). Communities with lower baseline knowledge may be less likely to recognize zoonotic risks associated with animal illness, carcass handling, slaughtering, hunting, animal bites, consumption of unsafe animal products, or contact with wildlife. In such areas, tailored interventions are likely to be more effective than generalized messaging, especially where literacy levels, language, cultural practices, or access to formal health information vary between communities (22). Age was a significant factor. Adult respondents had significantly higher adjusted odds of good knowledge compared with elderly respondents. This may indicate that adults are more exposed to formal education, recent sensitization activities, community meetings or occupational networks through which health information is shared. In contrast, elderly respondents may rely more heavily on long-standing traditional knowledge systems and may have less access to newer public health messages. This finding is consistent with previous studies, although the direction of association may vary by setting (34, 42). No significant differences were observed between genders, contrasting with previous studies that reported men exhibited slightly higher awareness, often attributed to their predominant roles in livestock handling and greater access to information (22, 33). This absence of statistical significance may reflect differences in exposure pathways. Men may interact through hunting and forest work, women through childcare, food preparation, and rearing small animals. It may also indicate equal reach of prior awareness campaigns. Education level was not significantly associated with knowledge. This result may appear unexpected, as formal education is often associated with better health knowledge (32). This may be explained by informal learning through experience, family, local folklore, accessible media, or prior disease outbreaks, narrowing the knowledge gap. The small number of highly educated respondents (only four with tertiary education, who scored low) limits the ability to draw definitive conclusions.

Although the training and awareness programs were associated with slightly higher odds of good knowledge, these associations were not statistically significant. This may indicate that the sensitization as captured in the dataset, was insufficient in intensity, duration, coverage, or follow-up to produce a measurable population-level shift in knowledge. Alternatively, the study may not have had sufficient statistical power to detect modest differences between pre- and post-assessment groups.

The coexistence of modern and traditional paradigms is common in African health contexts (43). Many people do not see them as opposites but, instead, as different complementary options. About 58.2% of Africans first seek treatment from traditional healers when they are sick (43). They do so because healers are located in close proximity, respected, and affordable (43). Even during severe outbreaks, people still use traditional remedies; for example, nearly half of Ebola survivors in Sierra Leone used them along with or before modern medicine (43).

This study revealed positive attitudes toward zoonotic disease prevention among respondents, who frequently expressed willingness to avoid sick animals, report unusual events, and seek medical care. However, these attitudes often contradicted actual practices. Similar knowledge, attitude, and practice gaps have been documented in Ghana (44), Vietnam (26), and Bangladesh (45, 46). In these regions, economic constraints, cultural traditions, and limited access to veterinary or health services hinder the translation of knowledge into safer behaviors (43, 47). Despite favorable knowledge and attitudes, risky practices remain widespread. In Mulundu, behaviors such as handling sick or dead animals, consuming meat from unknown sources, and failing to recognize early illness symptoms are common. Comparable risk patterns exist internationally; for example, livestock farmers in Ethiopia (25, 34), Rwanda (22), and India (2) often neglect testing animals for brucellosis or bovine tuberculosis. Protective equipment use is minimal, and high-risk behaviors, such as salvage butchering or barehanded handling abortive materials, are widespread. These findings highlight a persistent global challenge: knowledge does not inherently lead to safer behavior, especially when poverty, food insecurity, and cultural norms influence daily decisions (48).

This study is not without limitations. First, because the study used a cross-sectional design, it cannot establish temporal relationships and therefore does not support causal inferences about factors associated with knowledge or about the effects of sensitization activities. Second, the final sample size of 326 participants was lower than the minimum required sample size of 379 estimated for the study. This shortfall may have introduced selection bias, particularly if individuals who were more available, more engaged with community activities, or more interested in zoonotic disease issues were more likely to participate. In addition, the reduced sample size may have limited statistical power to detect modest differences between subgroups. Third, pre- and post-sensitization records were not fully matched at the individual level and were analyzed as independent observations. This may have diluted any true effect of sensitization or confounded differences between assessment phases through changes in participant composition across rounds.

## Conclusion

This study shows that although most participants in rural hunting communities of Mulundu Department had heard of zoonotic diseases, overall knowledge remained limited, particularly regarding specific zoonoses, animal reservoirs, indirect transmission routes, and clinical signs in humans and animals. Most respondents were classified as having poor knowledge, and important geographic disparities were observed, with participants from Regroupement A showing significantly lower odds of good knowledge than those from Regroupement B. Adults were more likely to have good knowledge than elderly respondents, suggesting that age- and community-specific communication strategies may be needed.

The findings also reveal a persistent gap between awareness, attitudes, and preventive practices. Many participants expressed willingness to seek hospital care, avoid sick or dead animals, or report unusual events. However, uncertainty in high-risk situations and continued reliance on traditional or spiritual forms of protection indicate that health messages must be culturally grounded and practically applicable. The absence of a statistically significant difference between pre- and post-training knowledge levels further suggests that one-off or short-term sensitization activities may be insufficient to achieve measurable and sustained behavioral change. Effective zoonotic disease prevention in rural Gabon will require continuous, participatory, and locally adapted One Health interventions that account for hunting practices, bushmeat consumption, and wildlife trade.

## Data Availability

The data generated during the current study are available from the corresponding author upon reasonable request.

## Supplementary material

Supplementary Material S1 Questionnaire administered during the study.
