# Supplementary material for: Zoonotic disease knowledge attitude and practices among rural hunting communities in Gabon
Source: Front Vet Sci. 2026 Jul 3;13:1857652. doi: 10.3389/fvets.2026.1857652 (PMC13375579; doi:10.3389/fvets.2026.1857652)
Supplement: Supplementary Material S1 — Questionnaire administered during the study. [file Table_1.docx]

**CENTRE INTERDISCIPLINAIRE DE RECHERCHE MEDICALES DE FRANCEVILLE**

**GABON**

**Supplementary Material**

**Questionnaire on zoonotic disease knowledge, attitudes, and practices among rural hunting communities in Gabon**

Dear participant,

This questionnaire was designed to collect data for the study entitled “**Zoonotic disease knowledge, attitudes, and practices among rural hunting communities in Gabon**”, conducted by the Centre Interdisciplinaire de Recherches Medicales de Franceville (CIRMF), Franceville, Gabon. The purpose of this study is to assess how people interact with animals and their environment, what they know about zoonotic diseases, and how they protect themselves and their communities. The information collected will help improve disease-prevention strategies, public-health programs, and community-based One Health activities in the study area.

Participation is voluntary. Participants may refuse to answer any question or stop the interview at any time without consequences. All information provided will be kept confidential and used only for research purposes. No personal identifiers will be recorded or shared. Participants are kindly asked to answer all questions as honestly as possible.

# Preliminary information

| **Item** | **Response** |
| --- | --- |
| Questionnaire code | __________________ |
| Interviewer name | __________________ |
| Regroupement | __________________ |
| Village | __________________ |
| Date of interview (dd/mm/yyyy) | __________________ |
| Assessment period | ☐ Pre-training / baseline ☐ Post-training / follow-up |

# Section 1. Socio-demographic information

| **Code** | **Question** | **Response options** |
| --- | --- | --- |
| S1 | Age in years | ________ |
| S2 | Gender | ☐ Male ☐ Female |
| S3 | Level of education | ☐ Illiterate ☐ Primary ☐ Secondary ☐ University |
| S4 | Main occupation | ☐ Farmer ☐ Hunter ☐ Salaried worker ☐ Student ☐ Trader ☐ Healer ☐ Driver ☐ Job seeker ☐ Retired ☐ Other: __________ |

# Section 2A. Knowledge questions included in the zoonotic disease knowledge score

The following 13 binary questions were used to calculate the knowledge score. Each item was coded as 1 for a correct or positive knowledge response and 0 for an incorrect, negative, or “do not know” response. The total knowledge score was calculated by summing K1 to K13, giving a continuous score ranging from 0 to 13, with higher scores indicating greater knowledge of zoonotic diseases.

| **Item** | **Dataset variable** | **Knowledge question** | **Response options** | **Score coding** |
| --- | --- | --- | --- | --- |
| K1 | Hear_zoonotic_disease | Have you ever heard of zoonotic diseases, that is, diseases that can be transmitted between animals and humans? | ☐ Yes ☐ No ☐ Do not know | Yes = 1; No/Do not know = 0 |
| K2 | Hear_animals_host | Have you ever heard that animals can carry or host diseases that may infect humans? | ☐ Yes ☐ No ☐ Do not know | Yes = 1; No/Do not know = 0 |
| K3 | transmitted_scratches | Can zoonotic diseases be transmitted to humans through scratches from infected animals? | ☐ Yes ☐ No ☐ Do not know | Yes = 1; No/Do not know = 0 |
| K4 | transmitted_through_bites | Can zoonotic diseases be transmitted to humans through bites from infected animals? | ☐ Yes ☐ No ☐ Do not know | Yes = 1; No/Do not know = 0 |
| K5 | transmitted_licking | Can zoonotic diseases be transmitted to humans through licking by infected animals, especially if saliva contacts broken skin or mucous membranes? | ☐ Yes ☐ No ☐ Do not know | Yes = 1; No/Do not know = 0 |
| K6 | transmitted_handling | Can zoonotic diseases be transmitted when handling sick animals, dead animals, carcasses, blood, or body fluids? | ☐ Yes ☐ No ☐ Do not know | Yes = 1; No/Do not know = 0 |
| K7 | transmitted_Bushmeat | Can zoonotic diseases be transmitted during bushmeat hunting, butchering, preparation, or handling? | ☐ Yes ☐ No ☐ Do not know | Yes = 1; No/Do not know = 0 |
| K8 | transmitted_Animal_consumption | Can zoonotic diseases be transmitted by eating meat or organs from infected animals, especially if insufficiently cooked? | ☐ Yes ☐ No ☐ Do not know | Yes = 1; No/Do not know = 0 |
| K9 | transmitted_Contaminated_water | Can zoonotic diseases be transmitted through water contaminated by animals, animal waste, blood, or other animal fluids? | ☐ Yes ☐ No ☐ Do not know | Yes = 1; No/Do not know = 0 |
| K10 | transmitted_inhalation | Can zoonotic diseases be transmitted by breathing contaminated droplets, dust, aerosols, or particles from animals or their environment? | ☐ Yes ☐ No ☐ Do not know | Yes = 1; No/Do not know = 0 |
| K11 | Zoonotic_kill | Can zoonotic diseases cause severe illness or death in humans? | ☐ Yes ☐ No ☐ Do not know | Yes = 1; No/Do not know = 0 |
| K12 | Know_signs_human | Do you know signs or symptoms that may suggest zoonotic disease in humans? | ☐ Yes ☐ No ☐ Do not know | Yes = 1; No/Do not know = 0 |
| K13 | Know_signs_animals | Do you know signs that may suggest disease in animals, such as abnormal behavior, weakness, bleeding, wounds, or sudden death? | ☐ Yes ☐ No ☐ Do not know | Yes = 1; No/Do not know = 0 |

**Knowledge-score calculation:** Knowledge score = K1 + K2 + K3 + K4 + K5 + K6 + K7 + K8 + K9 + K10 + K11 + K12 + K13. Possible score: 0 to 13.

# Section 2B. Open-ended knowledge follow-up questions analyzed descriptively only

These open-ended questions were asked to characterize the content of participants’ knowledge. They were not added as additional items in the 13-point knowledge score.

| **Code** | **Follow-up question** | **Response** |
| --- | --- | --- |
| F1 | If yes to K1, which zoonotic diseases do you know? | Open-ended response: __________________________ |
| F2 | If yes to K2, which animals do you think can carry or transmit zoonotic diseases? | Open-ended response: __________________________ |
| F3 | If yes to K12, which signs or symptoms in humans do you know? | Open-ended response: __________________________ |
| F4 | If yes to K13, which signs of disease in animals do you know? | Open-ended response: __________________________ |

# Section 3. Attitude questions

The following questions assessed intended responses to zoonotic-disease risk scenarios. They were analyzed separately from the knowledge score.

| **Code** | **Attitude question** | **Response options** |
| --- | --- | --- |
| A1 | What action would you take if a member of your family showed clinical signs or symptoms of disease? | ☐ Go to hospital ☐ Alert authorities ☐ Avoid contact ☐ Quarantine ☐ Traditional medicine ☐ Go to church ☐ Do not know ☐ Other: ______ |
| A2 | What action would you take if an animal showed clinical signs or symptoms of disease? | ☐ Avoid contact ☐ Alert authorities ☐ Kill ☐ Treat ☐ Do nothing ☐ Go to hospital ☐ Quarantine ☐ Veterinary medicine ☐ Do not know ☐ Other: ______ |
| A3 | What action would you take if you encountered a dead animal? | ☐ Avoid contact ☐ Alert authorities ☐ Bury ☐ Eat ☐ Veterinary medicine ☐ Do not know ☐ Other: ______ |
| A4 | What action would you take if you developed clinical signs or symptoms of an animal-borne disease? | ☐ Go to hospital ☐ Alert authorities ☐ Traditional medicine ☐ Quarantine ☐ Avoid contact ☐ Go to church ☐ Do not know ☐ Other: ______ |

# Section 4. Practice and perception questions

The following questions assessed reported practices, beliefs, and perceived protective measures. They were analyzed separately from the knowledge score.

| **Code** | **Practice/perception question** | **Response options** |
| --- | --- | --- |
| P1 | Do you think that prayer can protect you from zoonotic diseases? | ☐ Yes ☐ No ☐ Do not know |
| P2 | Do you think that charms can protect you from zoonotic diseases? | ☐ Yes ☐ No ☐ Do not know |
| P3 | Do you think that traditional bathing can protect you from zoonotic diseases? | ☐ Yes ☐ No ☐ Do not know |
| P4 | Do you think that traditional medicine can protect you from zoonotic diseases? | ☐ Yes ☐ No ☐ Do not know |
| P5 | Do you think that modern medicine can protect you from zoonotic diseases? | ☐ Yes ☐ No ☐ Do not know |
